# Interleukin-13 receptor α2 is associated with poor prognosis in patients with gastric cancer after gastrectomy

**DOI:** 10.18632/oncotarget.10297

**Published:** 2016-06-25

**Authors:** Chao Lin, Hao Liu, Heng Zhang, Hongyong He, He Li, Zhenbin Shen, Jin Qin, Xinyu Qin, Jiejie Xu, Yihong Sun

**Affiliations:** ^1^ Department of General Surgery, Zhongshan Hospital, Fudan University, Shanghai, China; ^2^ Department of Biochemistry and Molecular Biology, School of Basic Medical Sciences, Fudan University, Shanghai, China

**Keywords:** gastric cancer, IL-13R-α2, overall survival, prognostic biomarker, nomogram

## Abstract

**Background:**

Interleukin-13 receptor α2 (IL-13Rα2) plays a vital role in the invasion and metastasis of various types of cancer, but its role in prognosis of patients with gastric cancer remains unknown. The aim of this study was to investigate the impact of IL-13Rα2 expression on the prognostic value in gastric cancer patients after surgery.

**Results:**

Increased expression of IL-13Rα2 in tumoral tissue was associated with decreased overall survival rate (*P* < 0.001). IL-13Rα2 expression was an independent prognostic indicator for gastric cancer (*P* < 0.001). Stratification analyses showed IL-13Rα2 expression could give some additional prognostic information in tumors of different stages, especially in advanced tumors. Integrating IL-13Rα2 expression with generated a better nomogram that was validated by the validation set to predict the 5-year overall survival.

**Methods:**

IL-13Rα2 expression was evaluated by tissue microarrays from 507 gastric cancer patients from two academic medical centers and statistically assessed for correlations with the clinical profiles and the prognosis of the patients with gastric cancer. The prognostic nomogram was designed to predict 5-year overall survival probability.

**Conclusions:**

IL-13Rα2 expression might be an independent prognostic factor for gastric cancer after surgical resection and could potentially be a high-priority therapeutic target. Incorporating IL-13Rα2 expression into the TNM staging system can provide a good prognostic model.

## INTRODUCTION

Gastric cancer is one of the most common malignancies in the world. It is the fourth most common cancer worldwide and ranks as the second leading cause of cancer-related deaths [1] with an estimated 951,600 new cases and 723,100 deaths occurring in 2012 [2]. Curative resection is still the main treatment for resectable gastric cancer patients. Patients with early stage gastric cancer in Japan gain a 5-year overall survival as high as 76% [3]. However, if patients are diagnosed at advanced stage, the 5-year survival rate decrease to 20% [4]. Currently, the TNM staging system, composed of depth of tumor invasion, lymph node metastasis and distant metastasis, is the major prognostic value in clinical management of patients with gastric cancer. However, the TNM staging system cannot provide completely accurate prognosis because it doesn't involve tumor microenvironment. As a result, quite a few advanced-stage patients remain stable for years, whereas some patients with early-stage gastric cancer progress rapidly [5]. Considering the unpredictable and complicated natural history of gastric cancer, exploration of molecular pathways, identification of prognostic factors, and incorporation of more sensitive and accurate molecules into the TNM stage are of the utmost importance.

IL-13Rα2, which could bind IL-13 with high affinity alone, is one of two receptor subunits of IL-13R complex. In contrast to IL-13Rα2, IL-13Rα1 requires IL-4Rα to form a productive complex for IL-13-induced signal transduction [6, 7]. Furthermore, IL-13R-α2 is thought to act primarily as a ‘decoy’ receptor, sequestering IL-13 from the IL-13R-a1/IL-4R-a complex, and thus inhibiting its function. In addition, IL-13Rα2 is overexpressed in a variety of human tumors such as oral squamous cell carcinoma [8], breast cancer [9], clear cell renal cell carcinoma [10], hepatic cell cancer [11], ovarian cancer [12] and colorectal cancer [13]. Previous study has demonstrated the signaling function of the IL-13Rα2 in the development of gastrointestinal fibrosis [14]. Furthermore studies have revealed that IL-13Rα2 mediates metastasis in breast [15] and ovarian cancer [16]. In addition, studies in animal models of glioma [17] and pancreatic ductal adenocarcinoma [18] have shown that IL-13Rα2 chain plays a critical biologic role in IL-13 cytotoxin-mediated therapy. However, its prognostic value in gastric cancer is still unknown. Thus, illumination of the significance of IL-13Rα2 expression in gastric cancer might provide some additional prognostic information other than the TNM staging system for a further risk stratification and provide guidance for a more precise treatment for gastric cancer patients.

In the study, we analyzed IL-13Rα2 expression by immunohistochemical analysis in gastric cancer clinical specimens and its correlation with clinicopathological characteristics. Furthermore, we constructed a predictive nomogram to assess the 5-year overall survival of the patients with gastric cancer after gastrectomy.

## RESULTS

### Relation between IL-13Rα2 expression and clinicopathological features

**Figure 1:**
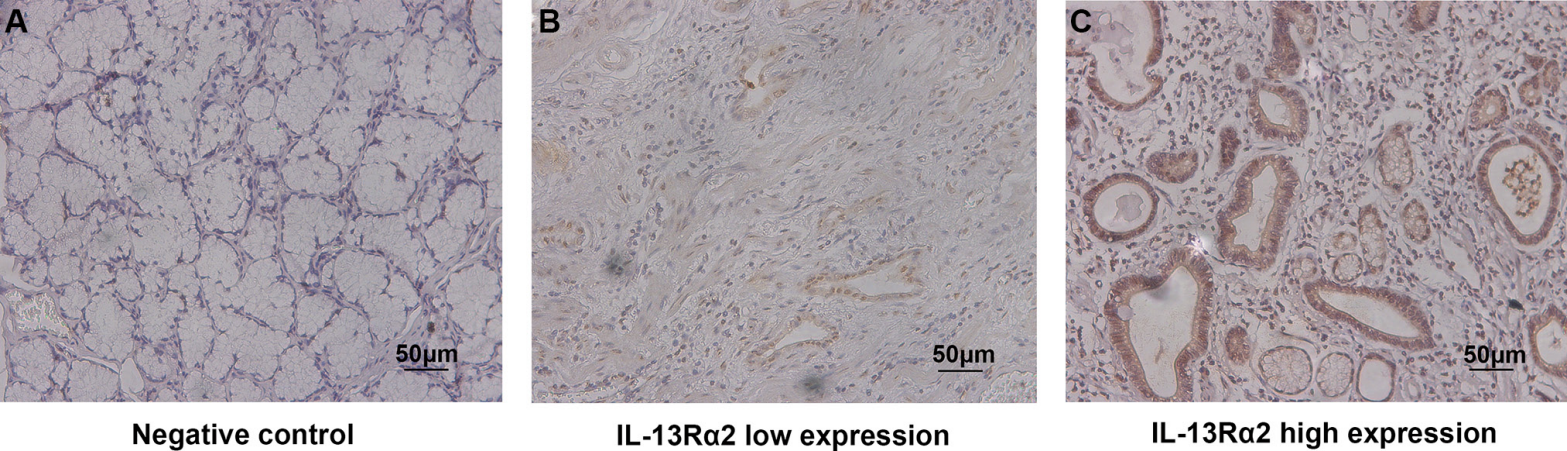
Expression of IL-13Rα2 in sections of gastric cancer Representative photographs of IL-13Rα2 expression (**A**–**C**). Negative control (A). Representative photographs of low and high expression of IL-13Rα2 (B–C). Original magnification: ×200.

Patients were divided into high and low IL-13Rα2 expression group according to the ‘minimum *P*-value method’ based on its relation with overall survival (cut-off value = 150). We evaluated the prognosis based on the expression of IL-13Rα2 in gastric cancer tissues. Figure 1A serves as the negative control. The expression of IL-13Rα2 was mainly localized in tumor cells (Figure 1B–1C). To evaluate the association of IL-13Rα2 expression with tumor biology, comparisons of the clinicopathological features with IL-13Rα2 expression were made. As shown in Table 1, IL-13Rα2 staining in training cohort was related to age (*P* = 0.029), depth of tumor invasion(*P* = 0.040), lymph node metastasis (*P* = 0.022) and TNM stage (*P* = 0.020). However, in the validation cohort, IL-13Rα2 expression was not associated with any clinicopathological feature. These heterogeneities may help to guarantee the predictor has universal application across heterogeneous population of patients in different regions.

**Table 1:** Associations between IL-13Rα2 expression and clinical pathological characteristics in patients with GC

Factor	Training set					Validation set				
	Patients		IL-13Rα2			Patients		IL-13Rα2		
	*N*	%	High	Low	*P*	*N*	%	High	Low	*P*
Age (years)					**0.029**					0.692
Mean ± SD	59.5 ± 11.7		60.8 ± 11.3	58.3 ± 12.0		65.3 ± 10.6		65.8 ± 10.5	64.9 ± 10.8	
Gender					0.605					0.802
Male	300	70.3	154	146		58	72.5	28	30	
Female	127	29.7	61	66		22	27.5	12	10	
Localization					0.237					0.850
Proximal	108	25.2	62	46		20	25.0	11	9	
Middle	60	14.1	29	31		28	35.0	14	14	
Distal	259	60.7	124	135		32	40.0	15	17	
Tumor size (cm)					0.687					0.453
Mean ± SD	3.9 ± 2.3		4.0 ± 2.3	3.9 ± 2.3		6.1 ± 2.8		6.4 ± 2.9	5.9 ± 2.7	
Differentiation					0.093					0.182
Well	20	4.7	8	12		1	1.3	0	1	
Moderately	158	37.0	90	68		26	32.5	11	15	
Poorly	249	58.3	117	132		53	66.2	29	24	
Depth of tumor invasion					**0.040**					0.249
T1	69	16.2	27	42		4	5.0	1	3	
T2	59	13.8	24	35		7	8.8	3	4	
T3	89	20.8	47	42		55	68.7	28	27	
T4	210	49.2	117	93		14	17.5	8	6	
Lymph node metastasis					**0.022**					0.232
N0	147	34.4	61	86		17	21.2	7	10	
N1	51	11.9	30	21		15	18.8	7	8	
N2	86	20.2	52	34		23	28.8	11	12	
N3	143	33.5	72	71		25	31.2	15	10	
Distant metastasis					0.426					1.000
M0	418	97.9	209	209		77	96.2	38	39	
M1	9	2.1	6	3		3	3.8	2	1	
TNM stage					**0.020**					0.241
I	94	22.0	38	56		5	6.2	1	4	
II	92	21.5	40	52		28	35.0	14	14	
III	232	54.4	131	101		44	55.0	23	21	
IV	9	2.1	6	3		3	3.8	2	1	

IL-13Rα2: Interleukin-13 receptor α2; SD: standard deviation;*χ^2^ test, Kruskal–Wallis test or Student's *t* test was performed. *P* < 0.05 was regarded as statistically significant.

### Prognostic significance of IL-13Rα2 for gastric cancer

**Figure 2:**
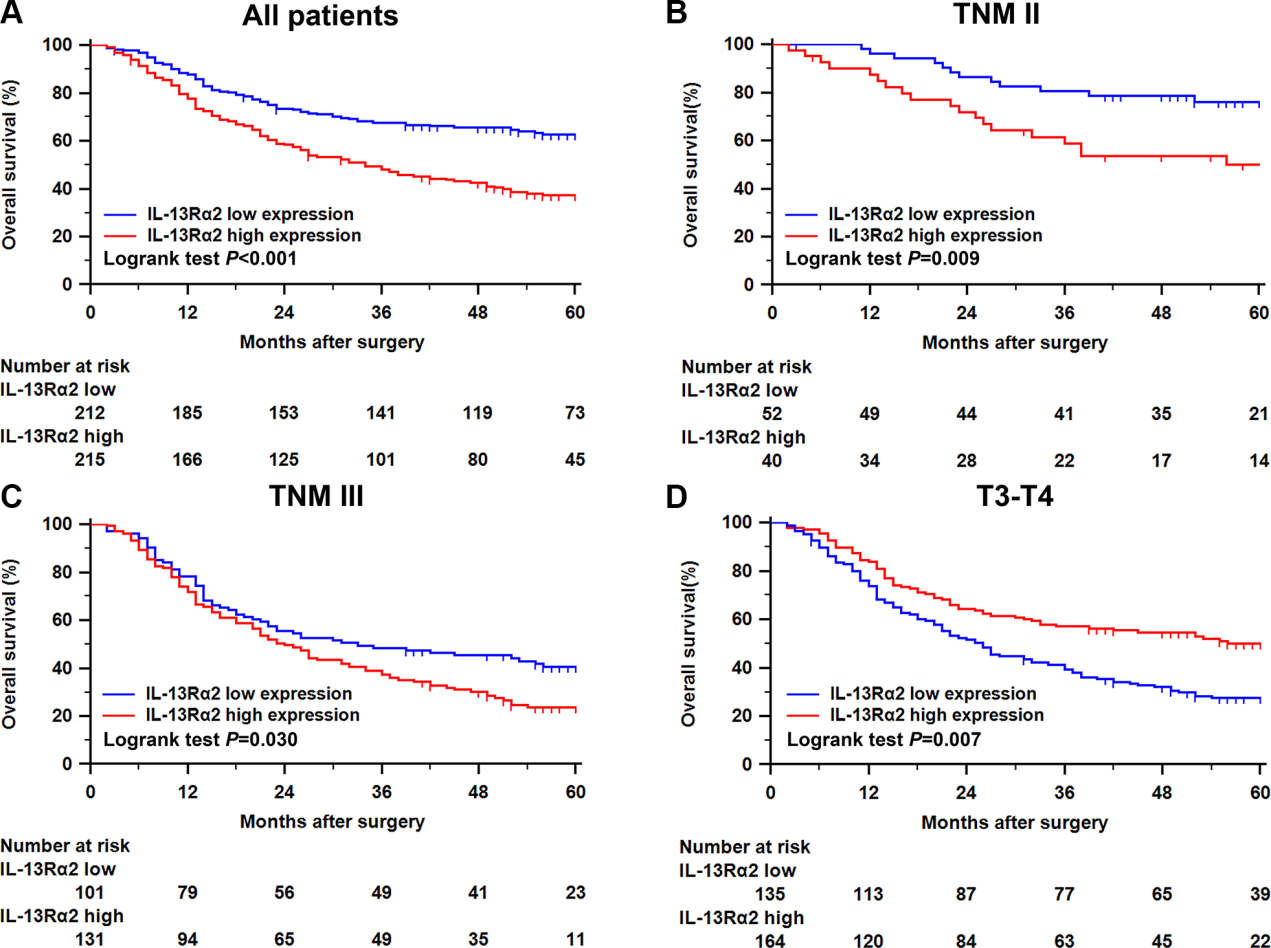
Kaplan–Meier analysis for OS of patients with gastric cancer according to the IL-13Rα2 expression Kaplan–Meier analysis for OS of patients with gastric cancer according to IL-13Rα2 expression in all patients (**A**), patients with TNM II stage tumor (**B**), patients with TNM III stage tumor (**C**), patients with T3-T4 stage tumor (**D**).

In order to estimate the clinical prognostic significance of IL-13Rα2 expression that might influence the overall survival of patients enrolled in this study, Kaplan-Meier survival analysis was performed in the training cohort. As shown in Figure 2A, patients with higher expression of IL-13Rα2 in tumor tissues were prone to lower OS. Low expression of IL-13Rα2 has a survival benefit compared with high expression (*P* < 0.001). Kaplan-Meier analysis was also applied to compare overall survival according to IL-13Rα2 expression in different TNM stage in tumor tissues. Significant difference was found in TNM II and III stage tumor according toIL-13Rα2 expression (Figure 2B, *P* = 0.009, Figure 2C, *P* = 0.030). To further explore the prognostic significance of IL-13Rα2 expression according to different clinicopathological factors, we performed Kaplan-Meier analysis in patients with different depth of tumor invasion. Significant differences were found in T3–T4 (Figure 2D, *P* = 0.007). In addition, Cox multivariate regression analyses were performed to define independent risk related to overall survival. As shown in the Table 2, depth of tumor invasion (HR, 1.28; 95% CI, 1.08–1.52; *P* = 0.004), lymph node metastasis (HR, 1.53; 95% CI, 1.33–1.76; *P* < 0.001), distant metastasis (HR, 2.55; 95% CI, 1.25–5.19; *P* = 0.009) and IL-13Rα2 expression (HR, 1.83; 95% CI, 1.38–2.43; *P* < 0.001) were all recognized as independent prognostic factors.

**Table 2:** Univariate and multivariate analyses of factors associated with survival in the training cohort

Factors	Univariate		Multivariate	
	H.R. (95% CI)	*P*	H.R. (95% CI)	*P*
Tumor size (cm)	0.90 (0.77–1.05)	0.224		
Differentiation	**0.71 (0.56–0.92)**	**0.009**	0.89 (0.68–1.15)	0.381
Depth of tumor invasion	**1.67 (1.44–1.94)**	**< 0.001**	**1.28 (1.08–1.52)**	**0.004**
Lymph node metastasis	**1.75 (1.52–1.94)**	**< 0.001**	**1.53 (1.33–1.76)**	**< 0.001**
Distant metastasis	**2.99 (1.48–6.07)**	**0.002**	**2.55 (1.25–5.19)**	**0.009**
IL-13Rα2	**1.95 (1.48–2.58)**	**< 0.001**	**1.83 (1.38–2.43)**	**< 0.001**

IL-13Rα2: Interleukin-13 receptor α2. *P* < 0.05 was regarded as statistically significant.

### Construction of the nomogram

**Figure 3:**
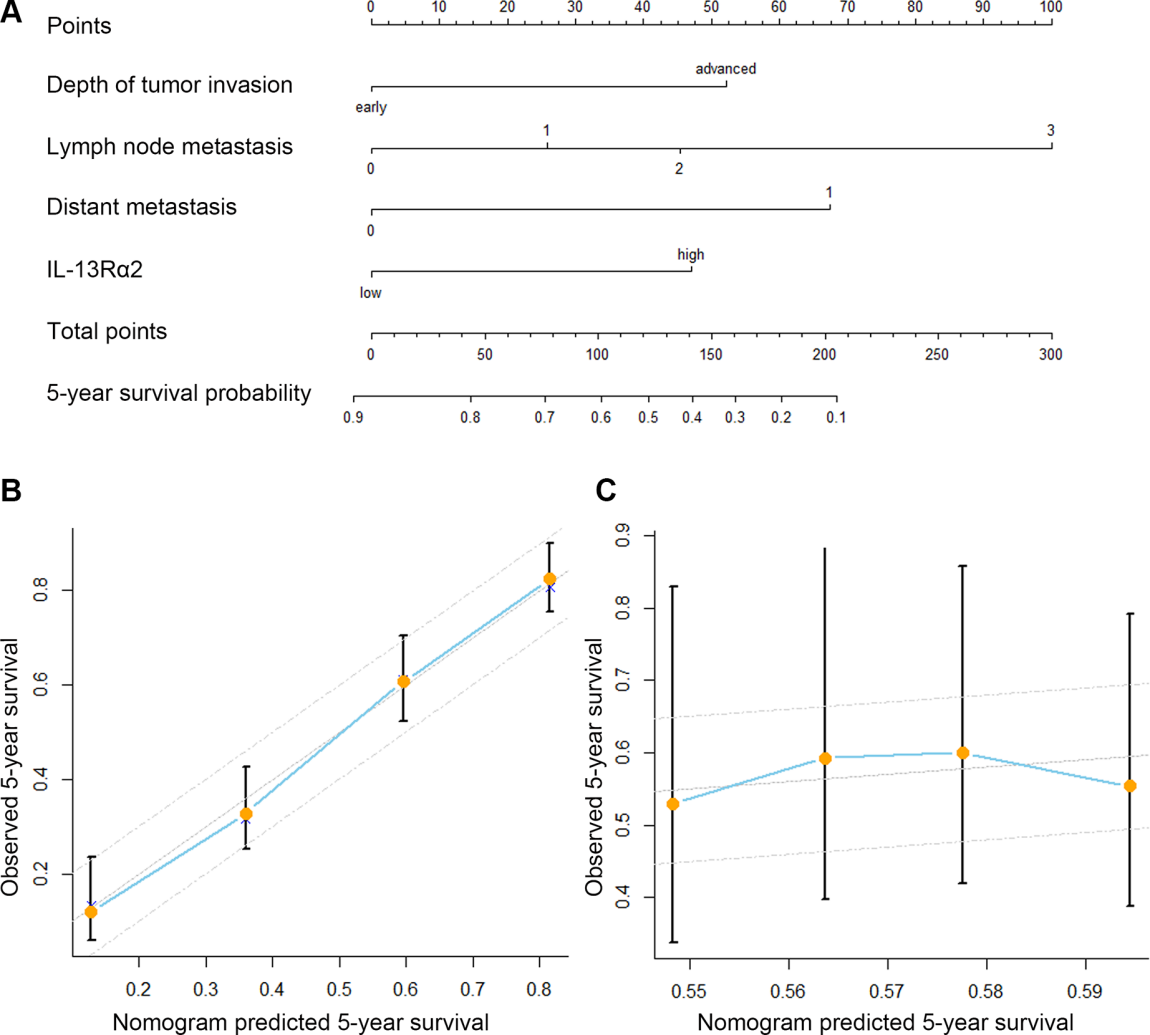
Prognostic nomogram generation for predicting overall survival in patients with gastric cancer (**A**) Nomogram for predicting postoperative 5-year survival probabilities after surgery, summing the score of the 4 variables, which is depth of tumor invasion (early/advanced), lymph node metastasis (N0/N1/N2/N3), and distant metastasis (absent/present), IL-13Rα2 expression (low/high). (**B**) Calibration of the nomogram for 5-year overall survival. Calibration curves for nomogram predicted 5-year overall survival corresponded well with the ideal model. Bars indicate 95% confidence intervals. (**C**) Caliberation of the nomogram in the validation set. The x-axis represents the nomogram-predicted survival, and the y-axis represents actual survival and 95% CI is measured by Kaplan-Meier analysis.

To predict the 5-year OS rates of gastric cancer, the following four independent variables, including IL-13Rα2 expression, depth of tumor invasion, and lymph node metastasis and distant metastasis status were selected in the nomogram (Figure 3A). The sum of the each variable point was plotted on the total point axis, and the estimated median 5-year survival rates were obtained by drawing a vertical line from the plotted total point axis straight down to the outcome axis. The calibration graph for the nomogram showed the probability of 5-year survival as predicted by the nomogram is plotted against the corresponding observed survival rates obtained by the Kaplan-Meier method (Figure 3B). The Harrell's concordance index (c-index) for the nomogram constructed by TNM and IL-13Rα2 expression was 0.657, higher than 0.632 of TNM alone. Figure 3C shows the calibration plot of the nomogram.

## DISCUSSION

The TNM stage system has been used as the prognostic factors for patients with gastric cancer. However, there are controversies about whether other risk factors, besides the TNM staging system, could be prognosticator for patients with gastric cancer. In the present study, 507 gastric cancer specimens were used to show the association between IL-13Rα2 expression and overall mortality after gastrectomy. In addition, high IL-13Rα2 expression was shown to be an independent poor prognostic factor independent of depth of tumor invasion, lymph node metastasis status, and distant metastasis status for OS. Furthermore, stratification analyses showed IL-13Rα2 expression could give some additional prognostic information in tumors of different stages, especially in advanced tumors. Hence, nomogram was constructed to predict the outcomes of the patients. The established nomogram showed better performance in predicting clinical outcomes for patients with gastric cancer after surgical resection than the TNM stage system alone. The nomogram was validated by the validation cohort.

As IL-13R is expressed in many cancers, IL-13 immunotoxins including IL-13PE were developed. IL-13PE could take effect in IL-13R positive RCC cells at a very low concentration [19]. Furthermore, IL-13PE was more cytotoxic in tumors with high level of IL-13Rα2. The cytotoxic role of IL-13PE to IL-13Rα2 expressed tumor was confirmed by plasmid-mediated gene transfer and knock-down experiments *in vitro* and *in vivo*. In addition to IL-13 immunotoxin, monoclonal antibodies have also been studied. Pandya et al. found that IL-13Rα2 monoclonal antibodies can aim at glioma tumors, delay their growth and improve the survival in animal models [20–21]. These results suggest that IL-13Rα2 antibody or a fusion protein may be a useful agent to target human cancer. However, the role of the IL-13Rα2 involved in gastric cancer remains far from being understood. Previously, Fujisawa et al. have demonstrated that IL-13 can activate IL13Rα2 to promote tumor invasion and metastasis by ERK/AP-1 pathway in animal model of ovarian cancer [16]. Meanwhile increasing evidence has shown that IL-13 contributes to transforming growth factor-beta activation by AP-1 pathway, which induces immunosuppression in patients with pancreatic cancer [22]. Moreover, elevated expression of IL-13 receptor α2 in colorectal cancer is related to liver metastasis and poor prognosis, suggesting IL-13 receptor α2 plays an important role in invasion, and metastatic colonization [23]. In the present study, we found the correlation between high expression of IL-13Rα2 and lymph node invasion. Furthermore, IL-13Rα2 could provide additional prognostic information in addition to the TNM stage system, especially in advanced tumors. Hence, it is reasonable for us to raise the hypothesis that IL-13Rα2 plays an important role in tumor progression and metastasis in gastric cancer.

IL13, as the ligand of IL-13Rα2, has numerous biological functions in inflammation and immune responses and potential clinical applications [24]. IL13 could be produce by T and B cells, and nature immune cells such as mast cells, natural killer and dendritic cells [25]. Gabitass and his colleague have also reported that IL-13 from myeloid-derived suppressor cells in pancreatic cancer could promote the tumor growth and metastases [26]. Since mast cell is an important source of IL-13 and some studies have reported that density of mast cells correlated with the presence of H. pylori in gastric neoplasia. What's more, it has been verified that mast cell correlates with lymph nodes metastasis in gastrointestinal cancer patients. In addition, Micu et al. found elevated concentration of mast cell has been shown to be related to poor prognosis in lung cancer [27]. As a result, we raised hypothesis that mast cells can secret IL-13 to promote metastasis via IL-13Rα2 in gastric cancer.

We revealed the prognostic value of IL-13Rα2 expression in gastric cancer, especially in more advanced tumors. However, a few limitations should be acknowledged. First, the overall survival rate differed between patients with IL-13Rα2 high and low expression in TNM IV stage tumors, mainly owing to a small number of patients with TNM IV stage. Second, the study was retrospective, meaning a large, multi-center, prospective research is needed to validate the results. In addition, Th-2 cells also secret IL-13, the predominant source of IL-13 in gastric cancer remains to be verified.

In conclusion, our study clearly have identified elevated expression of IL-13Rα2 was strongly associated with a poor outcome, which could be integrated with depth of tumor invasion, lymph node metastasis, and distant metastasis status to generate a nomogram to give a better risk stratification for gastric cancer patients with different prognosis, especially in more advanced stages.

## PATIENTS AND METHODS

### Patient selection

The study enrolled 507 patients with gastric cancer, of which 427 were from the Zhongshan Hospital, Fudan University (Shanghai, China) as a training cohort and the other 80 patients from Nantong Tumor Hospital (Jiangsu, China) represented the validation cohort. The study was approved by the two hospitals' research medical ethics committees, and written informed consent was obtained from each patient achieved. Radical gastrectomy with a D2 lymphadenectomy was performed on 495 patients between 2007 and 2008 while the surgery for the other twelve patients with distant metastasis was more focused on relieving symptoms. We retrospectively collected the clinicopathological and baseline demographic characteristics of the patients, including age, gender, tumor size, tumor differentiation, and tumor TNM stage. Tumor stage was reassessed according to the seventh edition of the UICC/AJCC TNM staging system. All the patients were followed up until April 2014. Overall survival (OS) was defined as the time from the date of surgery to the date of death or last visit.

### Tissue microarray and immunochemistry

Tissue microarray (TMA) construction and immunohistochemistry protocol were described previously [28], and the tissue microarray was established with formalin-fixed paraffin-embedded surgical specimens. The primary antibody against human IL-13Rα2 (Abcam, Cambridge, MA, USA) was applied in the procedure. Two pathologists blind to patient information evaluated the staining score of each specimen using the semi-quantitative immunoreactivity scoring (IRS) system [29], which was on a scale of 0–300, multiplying the percentage of positive tumor distribution (0–100 %) by the score of staining intensity (where 3, 2, 1, and 0 indicate strong, moderate, weak, and negative staining, respectively). The optimum cutoff score was 150 for the expression of IL-13Rα2 using X-tile software version3.6.1 (Yale University School of Medicine. New Haven. CT. 17 USA) based on the association with patients' OS. By X-tile software, we could tell the difference of OS between the IL-13Rα2 high group and IL-13Rα2 low group according to different cutoff value. When the cutoff value was set at 150, the difference was the most significant (*P* < 0.001).

### Statistical analyses

Analysis was performed with SPSS 21.0 (IBM Corporation, Armonk, NY, USA) and R software version 3.0.2 and the “rms” package (R Foundation for Statistical Computing, Vienna, Austria). Pearson χ^2^ test was used to compare categorical variables, and continuous variables were analyzed by Student's *t* test. The Kaplan-Meier method with log-rank test was used to compare survival curves. The Cox proportional hazards regression model was applied to perform univariate and multivariate analyses and those variables that achieved statistical significance in the univariate analysis were entered into the multivariable analysis. Furthermore, a nomogram was created by R software using “rms” package. Calibration plots were generated to examine the performance characteristics of the predictive nomogram. The Harrell's Concordance index (C-index) was used to quantify the predictive accuracy. All statistical tests were two-sided and performed at a significance level of 0.05.
